# In vivo biofunctional evaluation of hydrogels for disc regeneration

**DOI:** 10.1007/s00586-013-2998-8

**Published:** 2013-10-12

**Authors:** Sandra Reitmaier, Ludwika Kreja, Katharina Gruchenberg, Britta Kanter, Joana Silva-Correia, Joaquim Miguel Oliveira, Rui Luís Reis, Valeria Perugini, Matteo Santin, Anita Ignatius, Hans-Joachim Wilke

**Affiliations:** 1 Center of Musculoskeletal Research, Institute of Orthopaedic Research and Biomechanics, University of Ulm, Helmholtzstrasse 14, 89081 Ulm, Germany; 2 3B’s Research Group-Biomaterials, Biodegradables and Biomimetics, University of Minho, Headquarters of the European Institute of Excellence on Tissue Engineering and Regenerative Medicine, AvePark, S. Cláudio de Barco, Taipas, Guimarães, Portugal; 3 ICVS/3B’s-PT Government Associate Laboratory, Braga/Guimarães, Portugal; 4 School of Pharmacy and Biomolecular Sciences, University of Brighton, Brighton, UK

**Keywords:** Intervertebral disc, Degeneration, Regeneration, One-step, Sheep, Large animal model, In vivo

## Abstract

**Purpose:**

Regenerative strategies aim to restore the original biofunctionality of the intervertebral disc. Different biomaterials are available, which might support disc regeneration. In the present study, the prospects of success of two hydrogels functionalized with anti-angiogenic peptides and seeded with bone marrow derived mononuclear cells (BMC), respectively, were investigated in an ovine nucleotomy model.

**Methods:**

In a one-step procedure iliac crest aspirates were harvested and, subsequently, separated BMC were seeded on hydrogels and implanted into the ovine disc. For the cell-seeded approach a hyaluronic acid-based hydrogel was used. The anti-angiogenic potential of newly developed VEGF-blockers was investigated on ionically crosslinked metacrylated gellan gum hydrogels. Untreated discs served as nucleotomy controls. 24 adult merino sheep were used. After 6 weeks histological, after 12 weeks histological and biomechanical analyses were conducted.

**Results:**

Biomechanical tests revealed no differences between any of the implanted and nucleotomized discs. All implanted discs significantly degenerated compared to intact discs. In contrast, there was no marked difference between implanted and nucleotomized discs. In tendency, albeit not significant, degeneration score and disc height index deteriorated for all but not for the cell-seeded hydrogels from 6 to 12 weeks. Cell-seeded hydrogels slightly decelerated degeneration.

**Conclusions:**

None of the hydrogel configurations was able to regenerate biofunctionality of the intervertebral disc. This might presumably be caused by hydrogel extrusion. Great importance should be given to the development of annulus sealants, which effectively exploit the potential of (cell-seeded) hydrogels for biological disc regeneration and restoration of intervertebral disc functioning.

## Introduction

Intervertebral disc degeneration (IDD) is a highly relevant individual and socioeconomic burden, which is associated to various morphological and functional disturbances [1]. The gradual progression of the disease and the structural features, e.g., the degradation of proteoglycans with subsequent desiccation of the disc extracellular matrix, the ingrowth of blood vessels and the loss of intervertebral disc (IVD) height, can be addressed in close detail and predispose IDD for regenerative strategies [2].

Currently, different approaches are being pursued to regenerate the IVD. Direct injection of growth factors, viral vectors and cells, each alone or in combination, seek to stimulate proliferation and production of extracellular matrix [3]. Reasonable chances of success of these methods, however, are questionable [4, 5]. Low oxygen and pH within the avascular disc represent a hostile environment for cell metabolism [2, 6, 7]. Conversely, vascularization must not be promoted as it accelerates degeneration [8, 9]. Furthermore, aberrant mechanical stimuli may activate catabolic remodelling, cell death and tissue breakdown [10–13].

To reestablish a loading regime that enhances the anabolic response of resident and implanted cells and to trigger biological mechanisms of self-healing, biodegradable substitutes were designed according to the natural ideal of the IVD. As the restoration of disc height is assumed to be essential for nucleus replacements, sufficient quantities of hydrogels are intended to be injected into the IVD for an immediate restoration of disc mechanics. Biomaterial extrusion, however, is of major biomechanical concern [14–16]. Hyaluronic acid or polysaccharide-based hydrogels, such as gellan gum-based hydrogels, were proven to adequately support the growth and extracellular matrix deposition of cells and might therefore be suited as nucleus replacements [17–19]. To retain avascularity of cartilaginous tissues, functionalization of scaffolds with anti-angiogenic peptides was suggested [20].

To gain deeper knowledge on future research directions, the purpose of this study was to investigate newly developed hydrogels as nucleus replacements for the biomechanical restoration and biological regeneration of the disc. Different modifications of hydrogels were examined in an ovine nucleotomy model. The effect of functionalization on the efficiency of hydrogels was evaluated using anti-angiogenic peptides and bone marrow derived mononuclear cells.

## Materials and methods

24 adult Merino sheep (2–4.5 years; 76–108 kg) were operated to compare four different hydrogel configurations with nucleotomy controls. Permission for the animal experiment was received from the regional commission of Tübingen (Reg. Nr. 1032).

*Configuration 1* Hydrogels made of ionic crosslinked methacrylated Gellan Gum (iGG-MA; 3B’s Research Group, University of Minho, Portugal) were used as nucleus replacement. iGG-MA is a microbial polysaccharide, which forms a colloidal gel in the presence of metallic ions.

*Configuration 2* To investigate whether anti-angiogenic peptides reveal a positive effect in the prevention of IDD, iGG-MA was functionalized with non-cytotoxic polylysine-based VEGF-blockers (iGG-MA+PEP) that were shown to effectively inhibit endothelial cell proliferation [20] (School of Pharmacy and Biomolecular Sciences, University of Brighton, United Kingdom).

*Configuration 3* Hydrogels made of dodecyl-amide of hyaluronic acid (DDAHA; Anika Therapeutics, Abano Therme, Italy) were tested without additives.

*Configuration 4* For the cell-based approach, DDAHA was seeded with autologous bone marrow derived mononuclear cells (DDAHA+BMC). A one-step surgical procedure was performed combining the harvesting of bone marrow (BM) from the iliac crest, the isolation of BMC and the orthotopic implantation of DDAHA+BMC.

*Configuration 5* Nucleotomy control with no treatment.

The detailed composition of the hydrogels was previously described [21].

### Preparation of cell-seeded scaffolds

In 18 animals one of four operated IVD received a cell-seeded hydrogel. BM was harvested from the iliac crest under sterile conditions at the beginning of surgery. Coagulation was prevented by 5,000 IU heparin/10 ml BM (Heparin-Natrium-5,000-ratiopharm, ratiopharm GmbH, Ulm, Germany).

BMC (“buffy coat”) were isolated by buoyant density separation. The cell-seeded hydrogels were prepared by adding 0.25 ml of the cell suspension containing 4 × 10^6^ BMC/ml PBS to 0.5 ml of DDAHA, resulting in 1 × 10^6^ BMC/DDAHA. Control samples (DDAHA) were mixed with 0.25 ml PBS without cells. Both hydrogels were transferred in sterile 2 ml syringes for intraoperative application. For the estimation of the number of mesenchymal stem cells (MSC) in BMC a colony forming units-fibroblast (CFU-F) assay was performed as previously described [22].

### Orthotopic implantation of hydrogels

**Fig. 1:**
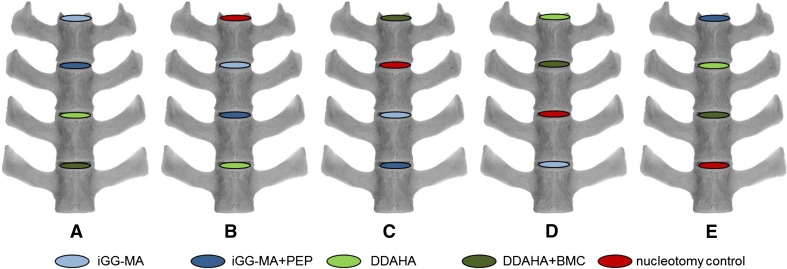
Alternating implantation scheme for the two hydrogels (*blue, green*) without (*light*) and with peptides or cells (*dark*) and the nucleotomy controls (*red*)

All animals were operated in four levels from L1–L2 to L4–L5 or in case of 7 lumbar vertebrae (*n* = 8) from L2–L3 to L5–L6. General anesthesia and the retroperitoneal multisegmental approach to the spine were performed as recently described in detail [23]. Each disc was stabbed with sterile microsurgery blades and a lateral nucleotomy was performed using 1.0 and 1.5 mm rongeurs with straight and flexed jaws, respectively. Approximately 0.20 g (0.17–0.23 g) nucleus tissue was removed from each disc and treated in an alternating sequence with one of the five configurations (Fig. 1). The annulus defects were closed with suture and 2-octyl-cyanoacrylate glue (DERMABOND^®^, Ethicon Products, Norderstedt, Germany) and covered with a collagen sponge (Lyostypt^®^, Aesculap AG, Tuttlingen, Germany).

Eight animals were sacrificed after 6 weeks for only histological and 16 sheep after 12 weeks for histological and biomechanical analyses. The 6-week group for biomechanics was omitted because after this time period annulus healing is not to be expected [24].

For biomechanical and histological comparison with intact discs, respectively, six native lumbar spines and six lumbar segments from independent comparable sheep were used.

### Biomechanics

For the biomechanical analyses, the polysegmental specimens including all treated discs were embedded in polymethylmethacrylate (PMMA, Technovit 3040, Heraeus Kulzer, Werheim, Germany) and tested in a custom-made spine loading simulator [25, 26]. The range of motion (RoM) and the neutral zone (NZ) were investigated for each segment separately at ±7.5 Nm pure moments in the three principal motion planes flexion + extension, lateral bending right + left, and axial rotation left + right using a motion tracking system (Vicon MX13, Vicon, Oxford, United Kingdom).

### Histology

For qualitative and semi-quantitative histological evaluation each segment was embedded in PMMA for undecalcified histology after formalin fixation. Giemsa staining was performed using standard protocols. An established semi-quantitative degeneration score according to Boos et al. [27] was used to determine the degree of degeneration (Table 1). The disc height index (DHI) was evaluated by dividing the mean value of the anterior, middle and posterior disc height from the histologic samples by the anterio-posterior diameter of the disc using ImageJ software [28]. Inflammation parameters, such as the presence of immune cells, regeneration of the tissue, blood vessel formation and remaining hydrogels were qualitatively evaluated under light microscopy (DMI6000B, Leica, Heerbrugg, Switzerland).

**Table 1:** Modified degeneration score according to Boos et al. [27]

Degeneration parameter	Characteristic feature	Score (total 36)
Intervertebral disc		
Cells	No proliferation/	0
	Increased cell density/	1
	Connection of two chondrocytes/	2
	Small size clones/	3
	Moderate size clones/	4
	Huge size clones	5
Granular changes	None/rare/intermediate/abundantly	0/1/2/3
Neovascularization	Absent/present	0/1
Rim lesions	None/rare/intermediate/abundantly	0/1/2/3
Concentric tears	None/rare/intermediate/abundantly	0/1/2/3
Radial tears	None/rare/intermediate/abundantly	0/1/2/3
Scar formation	Absent/present	0/1
Tissue defects	Absent/present	0/1
Endplate		
Cells	None/rare/intermediate/abundantly	0/1/2/3
Structural disorganization	None/rare/intermediate/abundantly	0/1/2/3
Clefts	None/rare/intermediate/abundantly	0/1/2/3
Microfracture	None/rare/intermediate/abundantly	0/1/2/3
Neovascularization	Absent/present	0/1
New bone formation	Absent/present	0/1
Scar formation	Absent/present	0/1
Tissue defects	Absent/present	0/1

### Statistics

For statistical analysis the unpaired, two-sample Wilcoxon signed rank test to a significance level of *p* ≤ 0.05 was used. Statistics were performed using GnuR [29].

## Results

Surgical interventions were well tolerated by the sheep. Intra- or post-operative complications did not occur.

### Cell isolation

The yields of BMC/ml BM and the frequency of MSC evaluated by CFU-F assay varied considerably between the sheep. The mean ± SD of BMC/ml BM was 1.11 × 10^6^ ± 0.95 × 10^6^. The cloning efficiency of BMC population varied between 0.0008–0.039 % CFU-F (mean 0.01 ± 0.01 %). This resulted in the mean of 101 ± 108 MSC/hydrogel and varied between 8 and 390 MSC/hydrogel. In average 0.20 ml (0.19–0.21 ml) of the cell-seeded hydrogels could be injected. The estimated number of BMC injected by DDAHA + BMC treatment therefore was about 270,000.

### Biomechanics

**Fig. 2:**
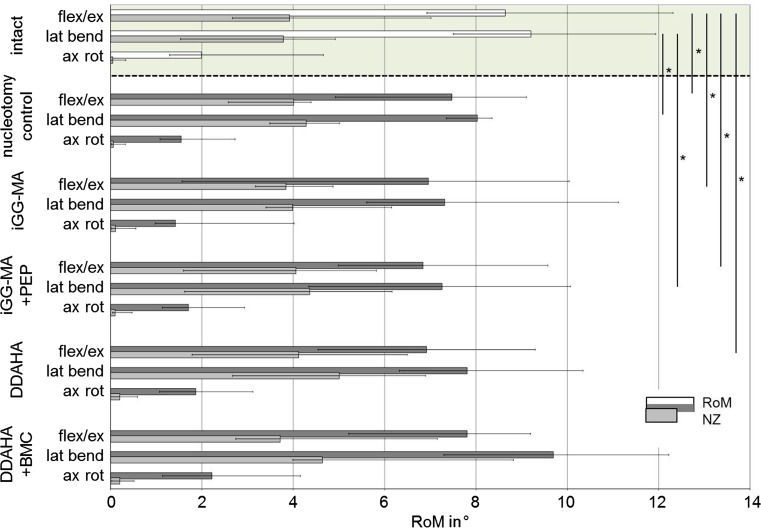
Total ranges of motion (RoM) and neutral zones (NZ) in flexion + extension (*flex/ex*), lateral bending right + left (*lat bend*) and axial rotation left + right (*ax rot*) for intact discs of separate sheep (*green background*) and for the five test configurations investigated in the current study. *p < 0.05

After 12 weeks in vivo, nucleotomy significantly decreased RoM compared to intact discs in flexion + extension (*p* = 0.02) and lateral bending (*p* = 0.04; Fig. 2), but not in axial rotation. Significant decreased RoM was also found for iGG-MA + PEP in flexion + extension (*p* = 0.03) and lateral bending (*p* = 0.02). RoM for iGG-MA and DDAHA was significantly lower only in flexion + extension (*p* = 0.04 and 0.01, respectively). DDAHA + BMC did not show significant differences at all. Compared to nucleotomy controls none of the hydrogel implanted discs revealed significant differences.

### Histology

**Fig. 3:**
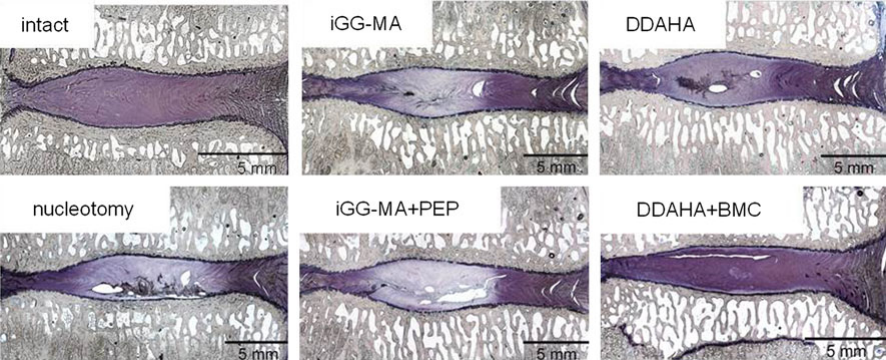
Representative mid-sagittal histological sections of operated discs 12 weeks after surgery in comparison to an intact disc

Histological sections showed marked differences between the surgically affected and intact discs 12 weeks after surgery. The structural integrity was obviously impaired in operated discs (Fig. 3). Tissue defects within the nucleus were similar in size in nucleotomy controls and hydrogel implanted discs. Hydrogels could not unambiguously be identified within the histological sections. Huge size cell clones were located in immediate vicinity of tissue defects inside the remaining nucleus pulposus tissue. Inflammation could not be found in any of the investigated samples. Marked neovascularisation did not occur in any of the operated discs and, therefore, differences between the hydrogel implanted discs and the nucleotomy controls were not found. No differences in cellularity were seen. There was no visible formation of spondylophytes or bony endplate changes up to 12 weeks.

**Fig. 4:**
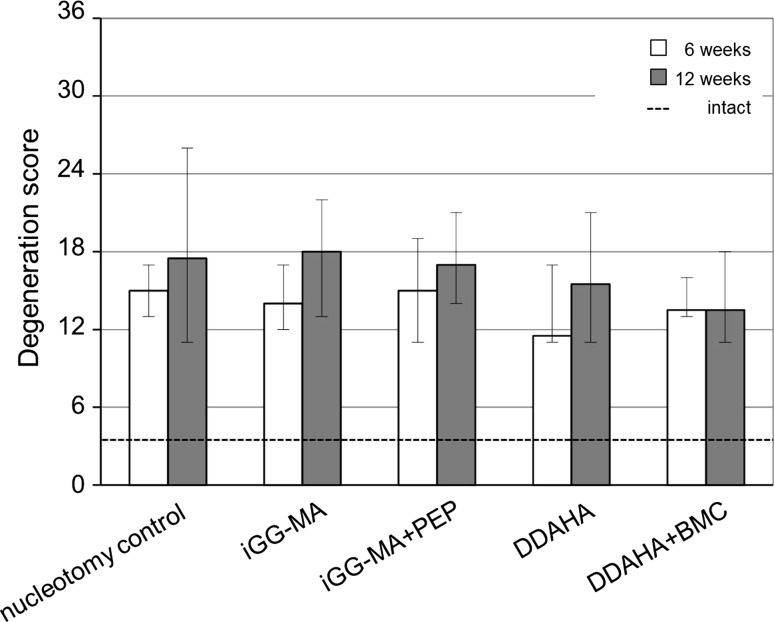
Degeneration scores of intact discs (*dashed line*) were significantly less than of nucleotomy controls and hydrogel implanted discs (*p* = 0.003 – 0.005). There was no difference between nucleotomy controls and the four different material configurations both after 6 (*white*) and 12 weeks (*grey*)

Intact discs revealed with a median degeneration score of 3.5 only negligible degenerative changes within the disc (Fig. 4). The degeneration score of all implanted and nucleotomized discs significantly differed from intact discs (*p* = 0.003–0.005). In contrast, there was no marked difference between the implanted discs and nucleotomy controls, neither after 6 nor 12 weeks. Comparing the results after 6 to results after 12 weeks, there was an obvious (although not significant) increase in the degeneration scores of nucleotomy controls (17 %) and all acellular test configurations (13–35 %). In DDAHA + BMC degeneration scores were kept almost constant.

**Fig. 5:**
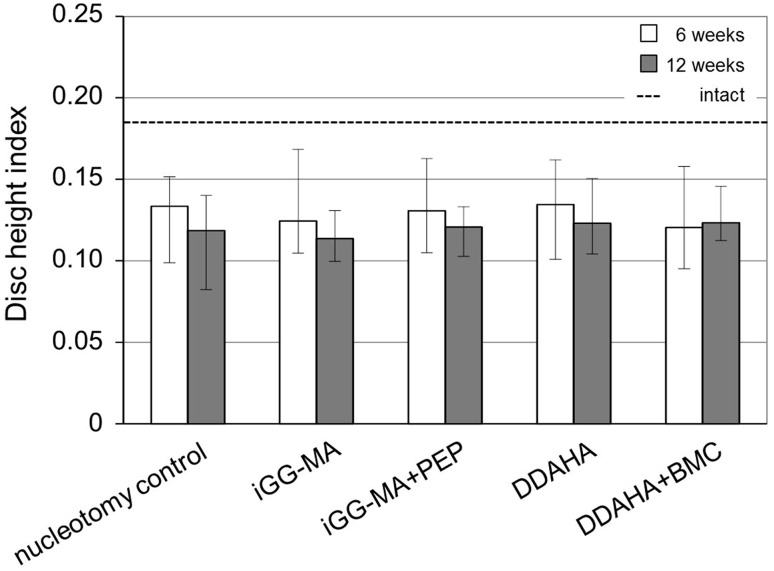
Disc height indices of intact discs (*dashed line*) significantly differed from nucleotomy controls and hydrogel implanted discs (*p* = 0.003 – 0.013). No difference between nucleotomy controls and the different material configurations was found both 6 (*white*) and 12 weeks (*grey*) post-operatively

DHI of intact discs were with 0.19 (0.16–0.25) significantly higher than all operated discs at both time intervals (Fig. 5). After 6 weeks DHI of nucleotomy controls fell by 28 % compared to intact discs. DHI of hydrogel implanted discs decreased in a similar range (iGG-MA: −33 %, iGG-MApep: −30 %, DDAHA: −27 %; DDAHA + BMC −35 %). From 6 to 12 weeks post-operatively there was a progressive decrease in DHI for acellular hydrogels (iGG-MA: −9 %, iGG-MApep: −8 %, DDAHA: −9 %). DHI in DDAHA + BMC, however, seemed to be preserved (+2 %) even if this effect was not significant. Nucleotomy controls lost 11 % of DHI between 6 and 12 weeks.

## Discussion

In this study using an ovine nucleotomy model, different configurations of newly developed hydrogels for nucleus replacements were investigated for their ability to restore RoM and disc height and to slow down IDD.

Results consistently showed that none of the tested hydrogel configurations proved to be superior to nucleotomy controls. The treatment with hydrogels was not able to restore disc height. However, against expectation, the operated discs showed a smaller ROM than intact discs. Histology of hydrogel implanted discs (acellular and cellular) showed more signs of degeneration as intact discs. There was a slight tendency that using BMC may eventually slow down IDD between 6 and 12 weeks of implantation.

The higher RoM of intact compared to nucleotomized discs found in this in vivo study is in contrast to in vitro data in literature, where RoM significantly increased after nucleotomy both for human [30] and for calf specimen [14, 31]. The same tendency was also found for ovine specimen in preliminary in vitro investigations for the current study (unpublished data). The discrepancy may be explained by general differences between in vitro conditions without healing, and the in vivo situation where collagenous bridges at the outer annulus were described 12 weeks after injury [32]. This initial bridging and the formation of scar tissue as well as the sealant method might be the reasons for the increased stiffness observed in this in vivo study.

The evaluation of the disc height index clearly proved that up to 12 weeks restoration of disc height could not be achieved using different hydrogel configurations as nucleus replacements after nucleotomy. This finding strongly indicates that relevant amounts of hydrogels were probably pressed out of the disc. This challenging problem of implant extrusion may also explain the similar disappointing degeneration scores both for the nucleotomy controls as well as for the hydrogel implanted discs.

Partial removal of nucleus was required for implantation of hydrogels as pure injection of a relevant amount of substitute materials into the healthy ovine disc is impossible because no cavity is available and intradiscal pressure in vivo is high [23]. Opening the annulus, however, inevitably causes the problem of extrusion. Sealants that reliably keep nucleus replacements inside the disc are urgently needed, but still not available. In previous in vitro studies, we have shown that the best of tested sealant options was cyanoacrylate glue combined with surgical suture [15]. Under axial compression, however, this sealant still allowed for gaping of the inner annulus defect with subsequent dislocation of implanted hydrogels and loss of intradiscal pressure in ovine motion segments [21]. To enhance the sealant efficiency in the current study, the closed defect was additionally covered with a collagen sponge. Artificial closure devices, proven to be effective to restore disc mechanics in vitro, unfortunately contradict to the main objective of disc regeneration and therefore were not used in the current study [33, 34].

Disc regeneration using hydrogels could not be achieved with this ovine nucleotomy model. This is in contrast to other animal experiments with rodents or pigs [35, 36]. These different findings may be explained by the persistence of notochordal progenitor cells within the discs of these animals. Because in humans and likewise in sheep this cell type disappears, conclusions from the above mentioned species should be transferred to humans with caution [37, 38]. Similar biology as well as similar anatomy and biomechanics suggest the sheep to be a more relevant model for humans [39–42].

The potential positive effect of BMC as additive in hydrogels for disc regeneration should be interpreted carefully. Only about 25 MSC/disc (range 2–70) provided in approximately 270,000 BMC have been implanted. The optimal number of bone marrow derived MSC to be implanted into the degenerated disc to achieve a stimulating effect was found to be 10^6^ in dogs [43]. However, MSC yields in this order of magnitude are impossible to achieve without in vitro expansion. Low cell density applied is assumed to be the reason for limited observed effects. In vitro expansion of BMC prior to surgery might have reinforced the trends. The nevertheless promising perspectives of cell-based approaches for disc regeneration are in accordance to literature [44, 45]. The right cell source, however, is still not finally clarified [46]. The BMC population used in the current study is a broad mixture of cell lineages in different differentiation stages containing multipotent mesenchymal and hemopoietic stem cells. Beneficial effects might be mediated by differentiation of MSC into IVD cells. Furthermore, BMC may act as trophic factor pools. Paracrine effects may cause BMC to secrete growth factors, cytokines and chemokines capable of stimulating the regeneration of injured tissue [47–49].

In case that the slight trend found in this study can be supported with future research, the perspective of applying non-cultivated BMC for disc regeneration in humans might be promising. Using the patients’ own and unmodified cells might be an interesting solution which would probably lead to higher compliance of this new potential therapeutic option [50–52].

Injected cells were not labelled in the current study. As with the hydrogels, the fate of BMC after injection is, therefore, not known. This limitation is owed to the one-step surgical procedure using BMC without in vitro cultivation.

The durations of the animal study with 6 and 12 weeks may eventually have been too short to represent the regenerative capacity of the different hydrogel configurations. Longer test durations are therefore recommended when investigating biological strategies.

### Conclusion

The results of present study indicate that because of presumed hydrogel extrusion, none of the investigated hydrogels was able to slow down IDD compared to nucleotomized discs. Therefore, the development of effective annulus sealants is crucial for successful nucleus replacement using hydrogels. Additional studies are recommended to substantiate the effects of non-cultivated cells for disc regeneration.
